# An introduction to real possibilities, indeterminism, and free will: three contingencies of the debate

**DOI:** 10.1007/s11229-018-1842-4

**Published:** 2018-06-09

**Authors:** Thomas Müller, Antje Rumberg, Verena Wagner

**Affiliations:** Department of Philosophy, University of Konstanz, P.O. Box 17, 78457 Konstanz, Germany

The papers collected in this Special Issue document the development of a novel, positive understanding of indeterminism grounded in real possibilities, and the interrelation of that development with the free will debate.

Our world is either deterministic or indeterministic, but not both. Given that the issue of determinism interacts with our understanding of freedom, it is important to understand what determinism and indeterminism amount to. Taking an understanding of determinism as given, one can characterize indeterminism purely negatively, as the negation of determinism, and leave it at that. In fact this seems to be the standard conception of indeterminism (cf. Butterfield 2005), and a positive characterization of indeterminism may seem hard to come by. Hacking (1990, p. 13) describes how the association of indeterminism (or chance) with fate or fortune led Hume and his followers to dismiss indeterminism as “the superstition of the vulgar” (Hacking 1990, p. 1). Strawson, in a classic of the free will debate, denounces indeterministic assumptions as “obscure and panicky metaphysics” (Strawson 1962, p. 25). Determinism, on the other hand, appears to be a solidly scientific notion: in the tradition of Laplace, determinism was purged of fatalistic connotations and grounded in hard facts about the state of the universe and the laws of nature. Most participants in the free will debate take the primacy of a positively characterized notion of determinism as the established doctrine. The same is true of the debate about deterministic versus indeterministic theories in the philosophy of science.

It is worth noting that the state of debate just sketched results from a historically contingent development within philosophy. In the sciences, indeterministic or statistic explanations have gained traction since the late nineteenth century. The link between non-deterministic scientific explanations and their metaphysical foundations is not straightforward. Some instances of indeterministic explanations are compatible with a deterministic metaphysics, pointing just to epistemic uncertainty with respect to a specific outcome. For example, when you buy a scratch card, you are most likely to win nothing. So if in fact you win nothing, you can refer to that statistical fact as an explanation. In this case, your use of statistics is merely epistemic: given the individual card, it is settled beforehand whether you will win or lose. It is just that your card, by design, provided no evidence for you to judge its true value. There are, however, clear cases of indeterministic explanations that do not build on an underlying deterministic base—consider explanations of the weather, stock markets, or the spread of an epidemic. And there are other areas of science in which base-level indeterminism is generally accepted or even used as a technological resource. In current evolutionary theory, for example, the idea of random mutation and successive selection can be phrased in terms of a positive understanding of indeterminism that is not in need of a deterministic complementation: the indeterministic exchange of one base for another in an organism’s DNA is taken as an explanatory unit. And in cryptography, physical randomness devices based on quantum indeterminism are acknowledged (and marketed) as vastly superior to other mechanisms for producing random numbers. The kind of indeterminism that is at stake here is as far removed from panicky notions of fate or fortune as one could wish: there is a clear mathematical formalism that describes the range of possible outcomes and their respective probabilities, and the whole theory is empirically thoroughly corroborated. For example, when a random bit is produced by letting a photon pass through a symmetrical beam splitter, the physical set-up allows for exactly two possible outcomes, with exactly 50:50 chance.

Given the development of a positively characterized notion of indeterminism in the sciences, a parallel approach in philosophy is a desideratum. A positive characterization of indeterminism seems especially important in the context of the current free will debate. In that debate, determinism as the metaphysical position that is characterized positively has a dialectical advantage: a positive characterization lends itself to theory construction, whereas a merely negatively characterized position is in the defensive. In order to be able to explore the possible role of indeterminism for an understanding of agency and freedom, we should try to characterize indeterminism in a positive way. The notion of *real possibility* marks one attempt at providing a positive philosophical characterization of indeterminism. In a nutshell, the idea is that indeterminism can be defined as the existence of multiple real possibilities for the future: an indeterministic world provides more than one option for how the future can unfold. Determinism is then just the negation of indeterminism, and is characterized as the lack of multiple real possibilities for the future. Arguably, this understanding of determinism makes explicit the tension with a notion of freedom that presupposes substantive alternatives. Explaining both determinism and indeterminism in terms of real possibilities furthermore shows that both notions are on an equal footing as far as their metaphysical basis is concerned. If one can spell out the notion of real possibility in a sensible way, there is no need to appeal to fate or fortune on either side.

In the next two sections we trace the way from real possibility to free agency via a positive characterization of indeterminism. Along the way we will point to two further contingencies in the respective debates, which add to the general point that a purely negative characterization of indeterminism is nothing but a historical contingency.

## Real possibilities and indeterminism

There are many different kinds of possibility, among which real possibility stands out in virtue of its peculiar interrelation with the notion of time. Next to epistemic possibility, we commonly distinguish, amongst others, between logical, metaphysical, and physical possibility. In contrast to real possibility, those latter kinds of possibilities, which are standardly discussed in the literature, are entirely atemporal notions. Epistemic possibilities as well as logical, metaphysical, and physical possibilities are generally conceived of as representing *modal* alternatives to actuality, i.e., ways our world could be in a certain respect but actually is not. Time enters the picture only in so far as actuality and its modal alternatives are usually considered extended in time. The term ‘possible worlds’ has become a synonym for possibilities thus understood. Each possible world stands in for some possibility, and one of them is the actual world, the world we actually live in, as we famously read in Lewis (1986). It is certainly right to say that the possible worlds framework has established itself as the standard approach to modality that is supposed to cover the entire variety of possibilities in a uniform way. This, however, is more of a historical contingency than a necessity. In our view, this second contingency is a byproduct of the widely held but fallacious belief that possibilities, in all their diversity, do not and cannot differ with respect to the relation they bear to actuality.

Real possibilities do not fit into the simple general scheme that underlies the possible worlds approach. In contradistinction to the standard notions of possibility, real possibility is inextricably interwoven with the notion of time, and the relation between actuality and possibility is a *temporal* rather than a modal one. Real possibilities are possibilities for the future. They are indexically anchored in concrete situations in time, and they are closely tied up with the world. What is really possible in a given situation is what can temporally evolve from that situation against the background of what the world is like. At the core of the notion of real possibility, there is the idea that—unlike the present and the past—the future is not actual yet. The future is yet to come, and real possibilities represent alternatives for that future to unfold. They are temporal alternatives *for* a dynamic actuality, so to say, rather than modal alternatives *to* a given actuality. Depending on whether the world is deterministic or indeterministic, in a concrete situation in time, there may be more than one possibility for the future, and each such possibility can be actualized. None of them is actual yet. Only as time progresses does one of them become actual, thereby ruling out the remainder. What is really possible then varies from time to time: in the course of time, the range of real possibilities diminishes.^1^

The difference between modal and temporal alternatives that is at stake here becomes most vivid if we consider a concrete example for each of the two paradigms. There is a sense in which it is possible that trees on earth grow more than 200 m into the sky. Just imagine a possible world in which the gravitational force is different from what it actually is. Such a possible world constitutes a modal alternative to our actual world, viz. one that is governed by different laws of nature. The notion of possibility at play here is an atemporal one, and it crucially differs from the one involved in the following example, which is an example for real possibility. Consider a radium atom—at a certain place, at a certain time. There are now two possibilities for the future: it is possible that the radium atom decays within the next century, and it is likewise possible that is does not. Those possibilities are not to be understood as mere epistemic possibilities that reflect our epistemic uncertainty with respect to what the future will bring—as was the case in the scratch card example. Rather, both scenarios in the radium example—decay or non-decay—constitute genuine temporal alternatives for the future: as of now, any one of them can be actualized. Unlike the example of trees growing 200 m into the sky, the radium example cannot be explicated in terms of possible worlds: neither of the future possibilities can be singled out as the actual one. From the standpoint of temporal actuality, there simply is no actual future.

Being tailored to the idea of modal alternatives, the possible worlds framework is suited to cover a broad range of different kinds of possibility. When it comes to the formal representation of real possibilities, however, the framework is of no avail. The peculiar interrelation of actuality, possibility, and time that is essential to the notion of real possibility requires a different formal setting. Real possibilities are most adequately pictured in theories of branching histories, such as the theory of branching time, pioneered by Prior (1967), or the theory of branching space-times, developed in Belnap (1992). In those theories, the modal-temporal structure of the world is depicted as a tree of histories that share some common past and branch toward the future. The tree-like representation gives expression to the idea that, at any given point in time, the past is fixed while there may be alternative possibilities for the future: real possibilities for what the future may bring.^2^

At the heart of the picture evoked by theories of branching histories, there is the idea of indeterminism as a positively characterized feature of the world, which we alluded to above. We said that, on a positive understanding, indeterminism can be characterized as the thesis that there is more than one real possibility for the future, whereas determinism is just the negation of indeterminism. With the framework of branching histories at our disposal, we can now make that claim more precise, and we can distinguish between local and global determinism and indeterminism. We can say that a situation is indeterministic if it corresponds to a branching point in the tree of histories. This provides us with a local notion of indeterminism. Here, indeterminism is defined from the perspective of a local standpoint in time: given the actual course of events up to now, there may be alternative possibilities for the future. A situation is accordingly dubbed deterministic if, locally, there is just a single possibility for actuality to evolve. A global notion of indeterminism can then be derived along the following lines: the world is indeterministic if the tree of histories that reflects the modal-temporal structure of the world contains at least one branching point. Consequently, the world can be indeterministic even though it is locally deterministic at some points. Global determinism, on the other hand, amounts to the complete absence of branching points in the modal-temporal structure of the world. In that case, the range of possibilities is restricted to a single history, which describes the only possible evolution of actuality. Indeterminism as defined here crucially differs from the standard definition of indeterminism as the mere negation of determinism: indeterminism has become a feature of our world itself. There is no need to refer to other possible worlds and their respective laws of nature.

While the above definition of indeterminism in terms of real possibilities makes no reference to possible worlds and their respective laws of nature, this by no means entails that the floor is open to mere chance. Given a concrete situation in time, against the background of what the world is like, it is certainly not the case that anything whatsoever can happen. The notion of real possibility goes hand in hand with a limited kind of indeterminism and thus presupposes laws of nature of some sort. In the possible worlds framework, laws of nature are usually associated with complete possible temporal developments. Real possibilities as temporal alternatives, on the other hand, require a different conception of laws of nature, viz. one that does not rely on the presence of an overarching actuality. One idea that suggests itself is to ground real possibilities in the nature of things. On such a view, what is really possible in a given situation is determined by what the objects can do in that particular situation in virtue of being the objects they are. Consider again our radium atom. The fact that this atom can possibly decay within the next century or not can be viewed as being rooted in the nature of radium. With respect to an atom of lead, no such real possibilities arise.

There are several recent strands of research that propose to ground possibilities in the potentialities or powers of the objects there are. Potentialities resp. powers are thereby conceived of as genuine modal properties of objects. What is characteristic of those properties is that they can be had by an object without being manifested. The manifestation constitutes a mere possibility, or so the idea goes. Potentialities or powers may give rise to different kinds of possibilities—temporal and atemporal ones—depending on whether one ties them to concrete situations in time or considers them in abstracto. The notion of real possibility requires that we take the former approach, i.e., we have to consider the possible manifestations of an object’s potentiality or power in a concrete situation. The basic idea can be phrased as follows: by manifesting their potentialities, objects become causally efficacious and jointly give direction to the possible future courses of events. In case our sample of radium consists of more than a single radium atom, there will, on the whole, be more than two possibilities for the future. Each radium atom of the sample can either decay within the next century or not, and every combination over those individual possibilities spans a possible course of events. As special cases, there will be one possible future continuation in which all radium atoms decay and another in which none of the radium atoms decays within the given time period.^3^

In explicating a positive understanding of indeterminism based on real possibilities, we have so far focused on what things in general can do in virtue of being the things they are, i.e., in virtue of their potentialities or powers. From there, it seems natural to move on to what *agents*—humans and other animals—can do in virtue of what they are, i.e., in virtue of their potentialities or powers. After all, there seems to be a close parallel between the fact that radium atoms can decay and trees can grow, on the one hand, and the fact that frogs can jump and crows can fly, on the other.^4^

## Indeterminism and free will

The free will debate is primarily concerned with agents and their actions. Yet, the term ‘free will’ was and still is used for many other notions that are either based on agency or situated in its direct neighbourhood. As a result, specialized discussions about very different notions such as moral responsibility, reason-responsiveness, rationality, personhood, autonomy, control, and others have emerged. At the heart of all those issues, however, there is the basic notion of agency and its metaphysics. Agents act within a physical environment: an environment that, for all we know, might be deterministic or indeterministic. Naturally, questions arise concerning the *compatibility* of agency or freedom with determinism and indeterminism. It turns out that the compatibility question regarding determinism and freedom has a different status in the debate than the compatibility question regarding indeterminism and freedom. We consider this asymmetry a third historical contingency that is closely connected to the first one, viz., the contingency that determinism is positively characterized while indeterminism is considered its mere negation.

The free will debate standardly has its focus on the consequences of determinism for free agency. This is obvious when we look at the major distinction in the free will debate. One might expect that that distinction concerns theories that affirm freedom versus those that deny it. However, the major distinction among free will theorists is whether they are *compatibilists* or *incompatibilists* with regard to freedom and determinism. Thus, the central question that splits the debate into two opposing parties is about the implications of determinism for freedom, but not about the implications of indeterminism.^5^

The consequences that this asymmetry has for the debate are significant: metaphysical implications of indeterminism are systematically not addressed and therefore invisible in the debate’s major distinction. Even though determinism and indeterminism are contradictories, answers to the compatibility question regarding freedom and determinism are logically independent from answers to the compatibility question regarding freedom and indeterminism. Consequently, traditional compatibilist or incompatibilist claims provide no verdict on the compatibility of freedom and indeterminism. A traditional compatibilist can be an indeterminism-compatibilist (neutral compatibilist) or an indeterminism-incompatibilist (soft determinist). Similarly, a traditional incompatibilist can be an indeterminism-compatibilist (libertarian) or an indeterminism-incompatibilist (hard incompatibilist).^6^

Despite being invisible in the debate’s major distinction, questions regarding the metaphysical implications of indeterminism are addressed in the form of two different side issues in the debate: the first one is a challenge to libertarian positions, while the second one concerns neutrality statements made by some compatibilists. For both of these side issues, the negative definition of indeterminism plays an important role.

In the free will debate, one comes across the compatibility question regarding freedom and indeterminism mainly in the context of the so-called luck objection, which is formulated as an incompatibility claim regarding agency and indeterminism. The luck objection suggests that, under indeterminism, actions are not attributable to the agent because indeterministic happenings cannot be under the agent’s control: they are a matter of ‘mere luck’.^7^ It is not always clear what the luck objection implies for the compatibilist who poses it. At least prima facie, a compatibilist who takes agency to be real, but incompatible with indeterminism, is committed to the truth of determinism. Most contemporary compatibilists, however, resist that strong claim, which raises questions about their metaphysical commitments. The luck objection is certainly a serious challenge for libertarians, especially for those who only argue that determinism is incompatible with free agency without offering a positive account of free agency under indeterminism. Nevertheless, compatibilists cannot just level this objection against their libertarian opponents without taking into account the consequences that the objection might have for their own position: such an attack may well fire back on their own compatibilist projects.^8^ Proponents of the luck objection can take advantage of a merely negative definition of indeterminism, which imposes no restrictions on what can happen, glossing indeterminism as ‘mere luck’, ‘anything goes’, or ‘acausality’. Such an unrestricted interpretation of indeterminism as the absence of action-attribution, of laws, or of causality makes the compatibilist position appear to be the more scientific or naturalistic one. As we pointed out in the beginning, this line of thought has contingent historical origins.

The second side issue of the debate that touches metaphysical implications of indeterminism concerns the most prevalent compatibilist position today, so-called neutral compatibilism. Again, a problem emerges from the merely negative definition of indeterminism, but in this case, indeterminism is played down rather than exaggerated. Compatibilists who aim at metaphysical neutrality vis-à-vis determinism exploit the fact that global determinism is already falsified by a single indeterministic occurrence on what they call the ‘microscopic level’. That level is supposed to have no or only a minor impact on agency, so that ‘macroscopic determinism’ could reign on the action-theoretic level. On such a view, it seems sufficient to provide a theory of agency under determinism.^9^ It is, however, at least doubtful whether the compatibility question regarding indeterminism is really addressed rather than avoided by this maneuver, and consequently, it may be asked to which extent the position in question is really metaphysically neutral.

The two side issues discussed above highlight the fact that a negative definition of indeterminism allows for extreme cases: determinism can be false in the sense that there is chaos and lack of restrictions everywhere, or in the sense that while the laws relevant for agency and daily life are deterministic, the world could be sprinkled with some inconsequential instances of indeterminism elsewhere. It seems time for discussions of indeterminism in the free will debate to fill the void between these two extremes. In our opinion, the best way of doing so is to spell out indeterminism positively, e.g., with the help of the aforementioned concept of ‘real possibility’ that provides a limited kind of indeterminism that is not insignificant on the action-theoretic level. This limited but not too narrow conception of indeterminism can be relevant for agency without neglecting causality and the natural order of the world. It is our hope to contribute to a turning point in the free will debate by providing room for theories that use indeterminism as a positive resource and break through the three contingencies that we have highlighted here.

## Contents of this Special Issue

This Special Issue brings together novel approaches that take different perspectives on a positive role of indeterminism in philosophy. Some papers favour a formal starting point, aiming at a clarification of the theoretical foundations of the debate; others take off from the phenomenon of agency, focusing on causation or on the role of alternatives. Yet others put metaphysics first, working at an elucidation of agency-related concepts via more basic notions such as powers.

On the formal side, Tomasz Placek’s paper provides an individuals-based account of the distinction between determinism and indeterminism of theories in philosophy of science (Placek 2016). Jan Broersen (2018) employs the formal resources of indeterministic branching time logic supplemented by representations of space in order to formulate a novel logic for agency in the tradition of stit (“seeing to it that”) theory. Peter Øhrstrøm (2016) discusses the origins of the formal analysis of indeterminism in Prior’s tense logic, showing the importance of considerations of human freedom for the formal treatment of real possibilities.

On the action-theoretic side, Ishtiyaque Haji (2016) and Erasmus Mayr (2017) consider the role of alternative possibilities in the debate about freedom and responsibility. Further contributions focus on the causal aspect of indeterminism and agency. While Laura Ekstrom (2016) aims at making sense of indeterminism in a strictly event-causal framework, Robert Kane (2016) points to the complexity of the debate and discusses different options for locating indeterministic processes in deliberation and action. The paper by Niels van Miltenburg and Dawa Ometto criticizes the wide-spread use of the causal theory of action in the free will debate, arguing for a new agent-causal approach (van Miltenburg and Ometto 2016).

On the metaphysical side, Ruth Groff (2016) argues that the free will debate should be rephrased in terms of powers and agency considered as strictly metaphysical notions. Barbara Vetter (2016), on the other hand, raises critical points about an analysis of abilities in terms of powers or dispositions.

## Footnotes

1 For a detailed discussion of the notion of real possibility and its relation to other kinds of possibilities, see Müller (2012) and Rumberg (2016b). In Rumberg (2016b, ch. 1), possibilities are differentiated with respect to the relation they bear to actuality, and the distinction between modal and temporal alternatives is introduced.

2 Theories of branching histories find their application primarily in modal-temporal logic [see, e.g., Thomason (1970), Zanardo (1998), and Rumberg (2016)]. They are also employed in debates about determinism and indeterminism in the philosophy of science [see, e.g., Müller (2015) and Müller and Placek (2018)]. Furthermore, they form the basis of the stit (“seeing to it that”) logic of agency [see, e.g., Belnap et al. (2001) and Broersen (2011)].

3 An account of real possibility in terms of potentialities along those lines is provided in Rumberg (2016b, ch. 4). Similar ideas can be found, e.g., in Prior (1962) and Vetter (2015).

4 For an account of (animal) agency based on powers, see, for example, Steward (2012).

5 On this point see also Müller and Briegel (2018).

6 Note that claims about compatibility or incompatibility are but modal claims regarding the *possibility* of free agents under the assumption of the truth or falsity of determinism. Without further premises, such claims neither imply that determinism is actually true or false, nor that free agency actually exists or does not exist. The only exception is the ‘hard incompatibilist’ position, which combines both incompatibility claims: the hard incompatibilist can infer the non-existence of free agents from the general impossibility of free agency (Strawson 1994). Given the logical independence of the two compatibility questions, however, the hard incompatibilist also has to argue for both incompatibility claims separately.

7 This objection is raised, e.g., by Hume (1748), Kane (1996), Van Inwagen (2000) and Mele (2006).

8 See Wagner (2013).

9 See, for example, Fischer (2007).
